# Clinical Protocol to Prevent Thrombogenic Effect of Liver-Derived Mesenchymal Cells for Cell-Based Therapies

**DOI:** 10.3390/cells8080846

**Published:** 2019-08-07

**Authors:** Louise Coppin, Mustapha Najimi, Julie Bodart, Marie-Sophie Rouchon, Patrick van der Smissen, Stéphane Eeckhoudt, Géraldine Dahlqvist, Diego Castanares-Zapatero, Mina Komuta, Sanne L. Brouns, Constance C. Baaten, Johan W. M. Heemskerk, Sandrine Horman, Nathalie Belmonte, Etienne Sokal, Xavier Stéphenne

**Affiliations:** 1 Laboratoire d’Hépatologie Pédiatrique et Thérapie Cellulaire, Unité PEDI, Institut de Recherche Expérimentale et Clinique, Université catholique de Louvain (UCLouvain), 1200 Brussels, Belgium; 2 Promethera Biosciences, 1435 Mont-Saint-Guibert, Belgium; 3 Unité CELL, Institut de Duve, Université catholique de Louvain (UCLouvain), 1200 Brussels, Belgium; 4 Unité d’Hémostase, Département des Laboratoires Cliniques, Cliniques Universitaires Saint-Luc, 1200 Brussels, Belgium; 5 Service d’Hépato-Gastroentérologie, Département de Médecine Interne, Cliniques Universitaires Saint-Luc, 1200 Brussels, Belgium; 6 Services des Soins Intensifs, Département de Médecine Aigue, Cliniques Universitaires Saint-Luc, 1200 Brussels, Belgium; 7 Pôle de Recherche Cardiovasculaire (CARD), Institut de Recherche Expérimentale et Clinique (IREC), Université catholique de Louvain (UCLouvain), 1200 Brussels, Belgium; 8 Service d’Anatomopathologie, Département des Laboratoires Cliniques, Cliniques Universitaires Saint-Luc, 1200 Brussels, Belgium; 9 Department of Biochemistry, Cardiovascular Research Institute Maastricht, University of Maastricht, 6211 LK Maastricht, The Netherlands; 10 Institute for Molecular Cardiovascular Research (IMCAR), University Hospital Aachen, RWTH Aachen University, 52074 Aachen, Germany

**Keywords:** cell- and tissue-based therapy, liver transplantation, mesenchymal stem cells, thrombosis, anticoagulants

## Abstract

The efficacy of mesenchymal stem cell infusion is currently tested in numerous clinical trials. However, therapy-induced thrombotic consequences have been reported in several patients. The aim of this study was to optimize protocols for heterologous human adult liver-derived progenitor cell (HHALPC) infusion, in order to eliminate acute thrombogenesis in liver-based metabolic or acute decompensated cirrhotic (ADC) patients. In rats, thrombotic effects were absent when HHALPCs were infused at low cell dose (5 × 10^6^ cells/kg), or at high cell dose (5 × 10^7^ cells/kg) when combined with anticoagulants. When HHALPCs were exposed to human blood in a whole blood perfusion assay, blocking of the tissue factor (TF) coagulation pathway suppressed fibrin generation and platelet activation. In a Chandler tubing loop model, HHALPCs induced less explosive activation of coagulation with blood from ADC patients, when compared to blood from healthy controls, without alterations in coagulation factor levels other than fibrinogen. These studies confirm a link between TF and thrombogenesis, when TF-expressing cells are exposed to human blood. This phenomenon however, could be controlled using either a low, or a high cell dose combined with anticoagulants. In clinical practice, this points to the suitability of a low HHALPC dose infusion to cirrhotic patients, provided that platelet and fibrinogen levels are monitored.

## 1. Introduction

Mesenchymal stem cell (MSC) infusion is an emerging therapy in regenerative medicine. Human MSCs from various tissues are currently under evaluation in numerous clinical trials. Of concern however, for clinicians and health authorities, is the emerging evidence for an increased risk of therapy-induced thrombosis reported in several patients receiving intravenous MSC infusions [1,2,3,4,5,6]. Mechanistic studies [7,8,9,10,11,12] have shown that infused MSCs frequently display procoagulant activity (PCA), linked to the presence of surface-expressed tissue factor (TF). However, a detailed characterization of the PCA of human liver-derived MSCs or heterologous human adult liver-derived progenitor cells (HHALPCs) is still lacking.

The link between TF expression and PCA was first described in patients receiving islet pancreatic cell transplantation [13], and was subsequently confirmed for hepatocytes [14,15] and MSCs from different origins, including for HHALPCs [7,8,9,16,17]. Surface-expressed TF is a known trigger of the coagulation cascade, leading to the conversion of prothrombin into thrombin traces, which in the presence of procoagulant membrane surfaces—such as provided by activated platelets—accumulate to form large amounts of thrombin and fibrin, resulting in a solid blood clot [18]. Studies with surface-immobilized TF have shown that expression numbers as low as one molecule of TF/μm^2^ are sufficient to stimulate the local formation of fibrin [19]. A TF-induced pathological activation of the coagulation cascade can result in a secondary depletion of platelets and coagulation factors, which may contribute to an increased hemorrhagic as well as thrombotic risk [20].

Infusions of HHALPCs are being developed as an advanced therapeutic medicinal product for the treatment of liver diseases. In particular, HHALPCs are considered for unstable patients with liver-based inherited metabolic diseases, needing orthotopic liver transplantation; as due to the shortage of donors, the risks of surgery, and the need for lifelong immunosuppression the utility of transplantation is limited. The therapeutic goal of repeated HHALPCs infusions is to engraft and mature into hepatocyte-like cells, to provide the missing enzyme(s) for long-term correction of the metabolic defect, which so far have been achieved only by transplantation of large cell masses of allogenic hepatocyte (5 × 10^7^ cells/kg or above) [3,21,22]. Additionally, due to their immunomodulatory [23] and anti-fibrotic effects [24], HHALPCs may also be used to treat acute decompensated cirrhotic (ADC) patients, in which transplantation of lower cell doses may be sufficient [25]. Disturbances of coagulation and platelet processes are however common in ADC patients. Concerns of thrombotic and/or hemorrhagic complications exist and could be induced by infusions of TF expressing cells [26,27]. 

The overall aim of this work was to study how HHALPC infusion can be performed safely with a minimal thrombogenic risk for metabolic or ADC patients. The consequences of a cell dose escalation were studied in a xenotransplant healthy rat model, in presence or absence of anticoagulants [17]. Furthermore, using a whole-blood flow model of HHALPC-induced thrombin and fibrin formation, the procoagulant role of expressed TF was evaluated. Finally, with a Chandler tubing loop model, the longer-term coagulation induced by HHALPCs was studied using human control and ADC blood.

## 2. Materials and Methods

### 2.1. Stem Cell Preparation

Heterologous human adult liver-derived progenitor cells (HHALPCs) were obtained from healthy donors (aged 3 days to 54 years) as previously described in the literature [28]. Cells used were cryopreserved at passages four to six. After thawing, cell batches with a viability >85% (trypan blue exclusion assay) were suspended in phosphate-buffered saline (PBS) with or without un-fractioned heparin (Heparin Leo 5000 IU/mL) at concentrations of 10 or 300 IU/5 × 10^6^ cells, and kept at 4 °C. 

### 2.2. In Vitro Studies

#### 2.2.1. Ethical Considerations and Blood Collection

Whole blood was obtained from healthy volunteers or from ADC patients in accordance with the Declaration of Helsinki (ClinicalTrials.gov identifier NCT03632148). Written informed consent was obtained from all subjects, and the study was approved by the review board of the Ethical Committee of Cliniques Universitaires Saint-Luc from Brussels (entry no. 2017/23JUI/331). All donors were free from antiplatelet or therapeutic anticoagulant treatment for at least 2 weeks. Acute-on-chronic liver failure (ACLF) in ADC patients was assessed according to the CLIF-SOFA score [29]. For Chandler tubing loop experiments, blood was collected via an open system with a 19G needle into heparin coated tubes (Lab Site Heparin Coating kit, Corline, Uppsala, Sweden) without adding any additional anticoagulants. For whole blood perfusion experiments, blood was obtained by venous puncture in a syringe containing trisodium citrate 3.8% (dilution 1:9).

#### 2.2.2. Microfluidic Whole-Blood Model

To determine if surface-expressed TF plays a crucial role in the cell-dependent PCA, we applied a previously validated microfluidics flow perfusion model [30,31]. Herein, thrombus formation was determined under flow at a defined wall shear rate by perfusion of citrated whole blood over a glass coverslip covered with cultured HHALPCs stained with Hoechst 33342 (f.c. 1 µg/mL, Thermo-Fischer Scientific, Waltham, MA, USA) to stain nuclei (blue). The coverslip was placed in a transparent parallel-plate perfusion chamber (width 3 mm, depth 50 μm, length 300 mm). Immediately before use, blood samples were pre-labeled with Alexa fluor (AF)647-fibrinogen (7.5 μg/mL, Thermo Fischer Scientific) to detect fibrin(ogen) (red); and DiOC_6_ (0.5 μg/mL, Anaspec, Fremont, CA, USA) to stain platelets (green) [31]. To suppress the TF pathway, 1 μM inactive factor VII was added; to suppress the intrinsic pathway, 40 μg/mL corn trypsin inhibitor was used. Using two pulse-free micro-pumps (Model 11 Plus, 70-2212, Harvard, Holliston MA, USA) and a mixing tube, whole blood citrated samples were mixed with coagulation buffer to obtain physiological concentrations of free Ca^2+^ and Mg^2+^. Blood perfusion into the flow chamber was at a wall-shear rate of 1000 s^−1^ for 6 min. Brightfield and fluorescence images were recorded every minute by an EVOS microscope, equipped with a 60× objective [32].

#### 2.2.3. Chandler Tubing Loop Model

The Chandler tubing loop model is an in vitro whole blood coagulation model that mimics the blood flow circulation. Blood (7 mL), from healthy volunteers (n = 4) or ADC patients presenting with ACLF grade 1 (n = 6, Table S1) was transferred in each loop, made of polyvinyl chloride tubing (inner diameter 6.3 mm, length 390 mm) and coated with heparin (C H coated PVC tubing of Ø 6.35 mm, Corline, Uppsala, Sweden). Either PBS or HHALPCs (500,000 cells/7 mL, corresponding to 5 × 10^6^ cells/kg) with or without heparin (10 or 300 IU/5 × 10^6^ cells) and/or bivalirudin (10.6 µg/mL) was added as mentioned. Loops were closed with a connector. To generate sufficient blood flow, the devices were placed onto a platform rocker inside a 37 °C incubator and subjected to flow/stirring at 65 rpm/min. Samples were collected from each loop after 5 and 60 min. Total blood counts were analyzed by an automatic hematology analyzer (XN-10™ Sysmex, Kobe, Japan). Plasma was obtained after double centrifugation at 2500 *g* for 10 min, and stored at −80 °C. Levels of fibrinogen and coagulation factors II, V, VIII, X, protein C, S and antithrombin were measured on an automated blood coagulation analysis system (ACL TOP 700 Werfen, Bedford, MA, USA). Thrombin–antithrombin (TAT) complex levels were measured by an ELISA assay (Enzygnost TAT micro kit, Siemens Healthineers, Germany). Results (except for TAT) were normalized in comparison to those from the control tubing loops containing only PBS. Results were expressed as arbitrary units (AU).

#### 2.2.4. Immunohistochemistry

Cryostat sections from macroscopic blood clots, obtained by tubing loop and embedded in Tissue-Tek O.C.T (Sakura Finetek, Torrance, CA, USA), were fixed in 4% para-formaldehyde and 50% ethanol before analysis. Slides were stained for platelets with an anti-CD41 antibody (rabbit anti-human 1:250, ab134131, Abcam, Cambridge, United Kingdom), for thrombin (mouse anti-human 1:200, ab17199, Abcam), for cell nuclei with 4′,6-diamidino-2-phenylindole (DAPI) and for cells with cell tracker red (Red CMTPX Dye, C34552, Thermo-Fischer Scientific). Four channel images were sequentially collected by a Cell observer Spinning Disk microscope (Zeiss, Oberkochen, Germany), equipped with a Plan-Neofluar 40×/1.30 oil objective and excitation laser lines of 405, 488, 561 and 635 nm, and emission channels at 460/80, 520/35, 617/73 and 685/40 nm.

### 2.3. In Vivo Studies

#### 2.3.1. Ethical Considerations

All experiments were approved by the Ethical Committee of Animal Experimentation at the Faculty of Science and health, Université Catholique de Louvain (UCLouvain, Brussels, Belgium), Belgium (Ref: 2015/UCL/MD/02).

#### 2.3.2. Cell Transplantation

Anesthetized male Wistar Han rats (150–200 g) (n = 6/group) were transplanted in sterile conditions with one of three different cell dosages, 5 × 10^6^ cells/kg, 1.25 × 10^7^ cells/kg or 5 × 10^7^ cells/kg, by intraportal injection, with or without intravenous administration of bivalirudin (0.75 mg/kg Angiox, Medicines Company, NJ, USA). The cell dose range originated from clinical cases of patients transplanted with HHALPCs [21,33] or hepatocytes [34,35]. For safety studies, the lowest cell dose, 5 × 10^6^ cells/kg, was infused by intraportal and peripheral infusion. Rats were sacrificed after 1 h, and their livers were harvested and fixed in 4% formaldehyde. Blood samples were taken from the portal vein, both before and after transplantation, with a syringe containing trisodium citrate 3.8% (dilution 1:9). Total blood counts were analyzed by an automatic hematology analyzer (Cell-DYN Emerald, Abbott Diagnostics, IL, USA). Plasma was obtained by centrifugation at 2700 *g* for 15 min and stored at −80 °C. Levels of fibrinogen and coagulation factors II, V, VIII and X were measured on an automated blood coagulation analysis system (ACL TOP 700 Werfen, Bedford, MA, USA). Thrombin–antithrombin (TAT) complex levels were measured by an ELISA assay (Enzygnost TAT micro kit, Siemens Healthineers, Germany).

#### 2.3.3. Immunohistochemistry

Rat liver sections (5 μm thickness) were stained with hematoxylin eosin (HE) and phospho-tungstic acid hematoxylin (PTAH) for fibrin coloration, or immunostained with a human anti-β1 integrin antibody for HHALPC presence (Bioke #9699, 1:300). Stained slides were digitalized using a SCN400 slide scanner (Leica Biosystems, Wetzlar, Germany) at 20× magnification, and analyzed using the image analysis tool Author version 2017.1 6.9.2 (Visiopharm, Hørsholm, Denmark). Tissue sections were automatically surrounded, portal veins were manually delineated, and integrin-expressing cells were detected at high resolution (20×) using a thresholding classification method, based on preprocessing steps highlighting 3,3′-diaminobenzidine staining (Dako k4003, Glostrup, Denmark). Thresholds were adjusted for representative stained vs. non-stained regions. Parameters were kept constant for all slides [36,37].

#### 2.3.4. Intravital Microscopy

For intravital microscopy (IVM), we adapted the previously mentioned transplantation protocol. Fluorescent-labeled HHALPCs (CellTracker Red CMTPX Dye, C34552, Thermo-Fischer Scientific) at a higher dose of 5 × 10^7^ cells/kg were transplanted by intraportal injection into adult Wistar rats (n = 3/group). Liver vascularization was assessed at different time points (1 h, 24 h, 48 h and 7 days) after transplantation by intravital microscopy (S1 Supplementary Data). Liver vasculature and cell nuclei were stained by intravenous injection of 5 mg of 70kDa FITC-dextran (46945, Sigma, St-Louis, MO, USA) and 0.2 mg Hoechst 33342 (H1399, Invitrogen, Carlsbad, CA, USA). Images were collected using an LSM 510-NLO laser scanning microscope (Zeiss, Oberkochen, Germany) in multiphoton mode, equipped with a 25× LD-objective NA0.8. For fluorescence excitation, a laser line of 800 nm (2%) was used, and detection was through 3 emission channels (Metadetector window 585–628 nm for red emission; two bandpass filters BP 435–485 for blue, and BP 500–550 for green emission). Z-stacks were collected to reconstruct 3D images using Zen 2012 image processing software (Zeiss).

#### 2.3.5. Statistical Analysis

The Mann–Whitney or the Wilcoxon signed rank test was used (two tailed, 95% confidence intervals). P-values lower than 0.05 were considered statistically significant (* *p* < 0.05, ** *p* < 0.01, *** *p* < 0.001). Analysis was performed with GraphPad Prism version 8.0.1 for Windows (GraphPad Software, San Diego, CA, USA).

## 3. Results

### 3.1. Anticoagulant Drugs Suppress the Coagulation Induced by HHALPCs in Whole Blood In Vitro

**Figure 1:**
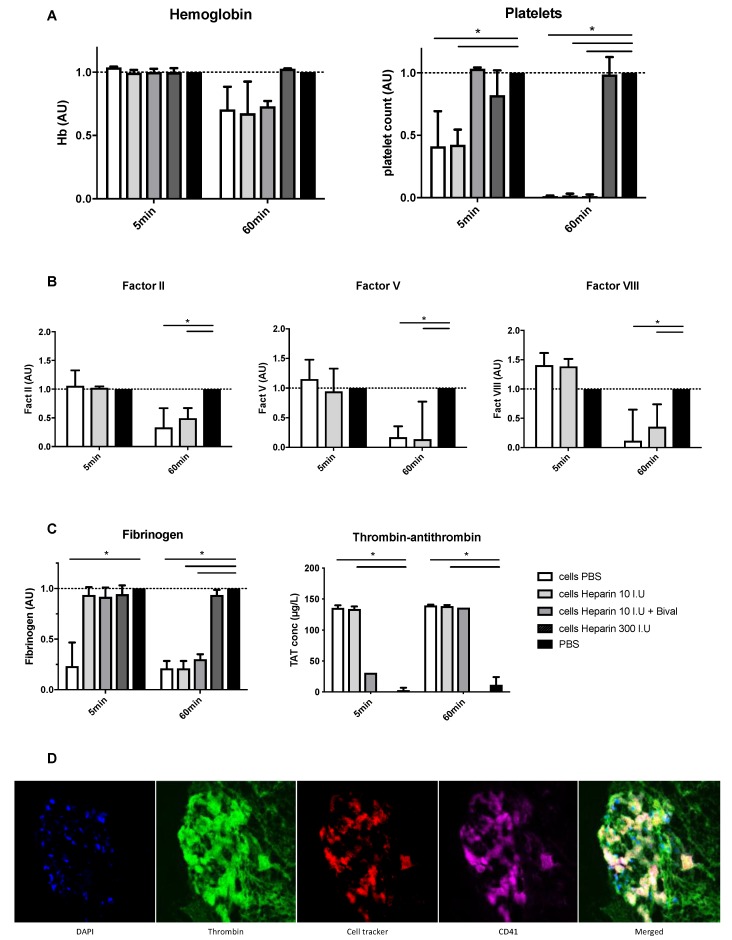
Heterologous human adult liver-derived progenitor cells (HHALPCs) were added to the tubing loops with or without anticoagulant drugs, such as low dose heparin (10 IU), heparin and bivalirudin, and high dose heparin (300 IU) as depicted. Blood samples were performed after 5 and 60 min. (**A**–**C**) All values, except for thrombin–antithrombin (TAT) complex levels, were normalized in comparison to control tubing loops containing only phosphate-buffered saline (PBS) (expression as arbitrary units (AU)). Note that adding bivalirudin or heparin interfered with the chronometric coagulation factor measurements, so no results of these conditions are represented. Bars represent medians with interquartile ranges (n = 4). Mann–Whitney test (* *p* < 0.05). (**D**) Immunofluorescence images of HHALPCs entrapped by thrombin and platelets in a blood clot retrieved after 60 min. Labeled HHALPCs (red) were localized at the core of the blood clot, surrounded by CD61^+^ (violet) platelets and entrapped in thrombin (green). Nuclei were counterstained on each image with DAPI (blue). Objective 40×.

Using the ex vivo Chandler tubing loop model, we characterized the procoagulant activity of HHALPCs with blood from healthy volunteers (n = 4) in the presence or absence of anticoagulants. Tubing loops of HHALPCs with or without anticoagulant drugs were inspected at 5 and 60 min after blood insertion. The presence of HHALPCs induced a significant decrease in platelet count, fibrinogen, factor II, V and VIII levels, and induced a significant increase in TAT complex levels (Figure 1). No change in hemoglobin level was observed and the factor X levels remained unchanged (Supplementary Data S2). This pointed to activation of coagulation by the HHALPCs that was suppressed by the addition of 300 IU heparin/5 × 10^6^ cells. Immunofluorescent staining of the retrieved blood clot after 60 min demonstrated labeled HHALPCs (red), entrapped by CD61^+^ platelets (violet) and thrombin (green) (Figure 1D). This supported the conclusion that the HHALPCs stimulated platelet activation and coagulation, forming a thrombus around the cells.

### 3.2. Coagulation Activation of HHALPCs by TF in a Shear Stress Model

**Figure 2:**
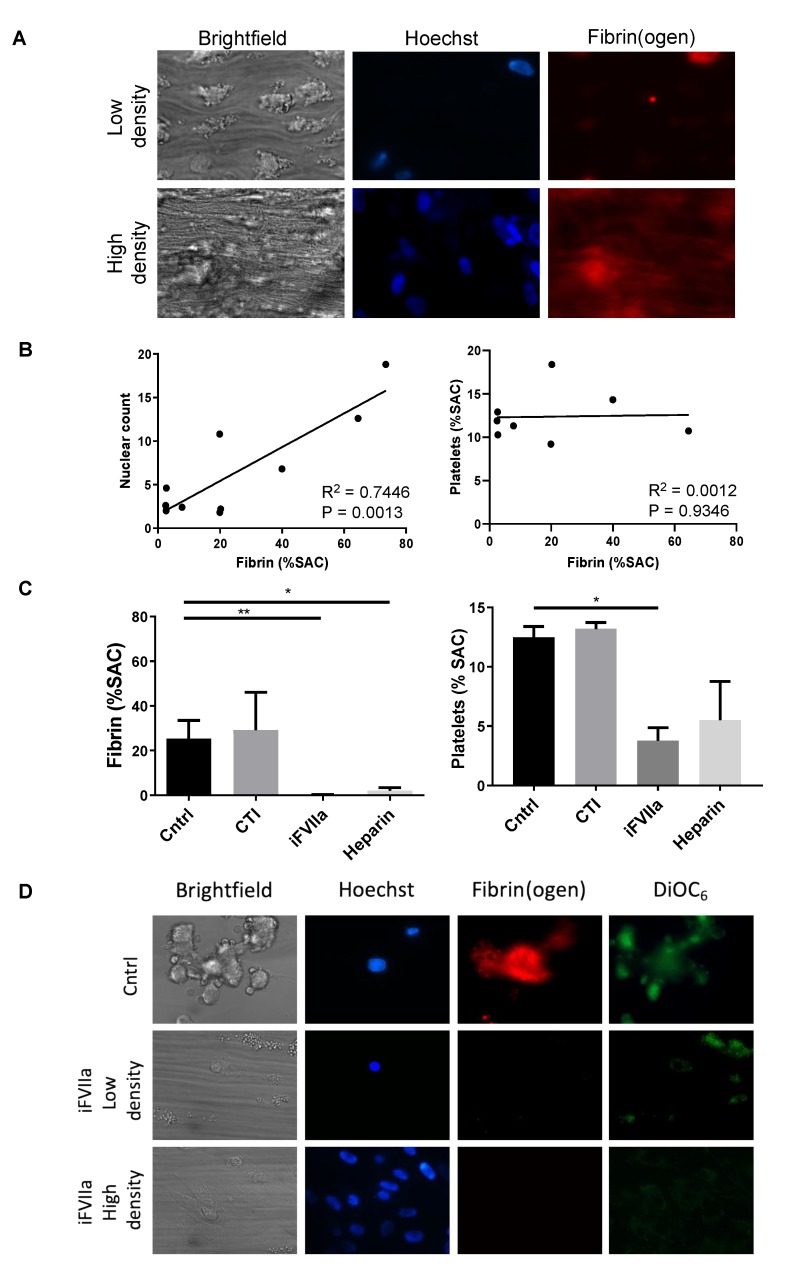
Recalcified citrate anticoagulated whole blood was perfused over HHALPCs with cells seeded at either low or high density. Microscopic images were taken after 6 min of blood perfusion. Cells were stained with Hoechst (blue) to visualize cell nuclei, while Alexa fluor (AF)647-fibrinogen (red) and DiOC_6_ (green) were used to measure fibrin formation and platelet activation, respectively. Fibrin surface area coverage (SAC) was analyzed using Fiji software. (**A**,**B**) Note a positive linear correlation (R^2^ = 0.7446, *p* = 0.0013) between cell density and fibrin generation. (**C**,**D**) Fibrin generation and platelet activation was significantly reduced when inactive FVIIa (iFVIIa) (*p* < 0.01) was supplemented. On the contrary, corn trypsin inhibitor (CTI) did not interfere with fibrin generation or platelet activation. Data are presented as mean + SEM, non-parametric test (Kruskal–Wallis) * *p* < 0.05, ** *p* < 0.01.

In recalcified whole-blood exposed to HHALPCs in a microfluidics device, we determined the activation of coagulation under high-shear flow conditions. Herein, it appeared that the extent of fibrin generation, and thus activation of coagulation, increased with the number of HHALPCs present (Figure 2A,B). No correlation was observed between platelet activation and fibrin generation. To evaluate the role of TF, the flow experiments were repeated with inactive factor VII, blocking the extrinsic pathway of the coagulation cascade [38]. A major decrease in fibrin generation and platelet activation was observed (Figure 2C,D). On the contrary, adding corn trypsin inhibitor to block the intrinsic pathway, did not interfere with fibrin generation or platelet activation. Moreover, adding heparin resulted in reduced coagulation with a significant decrease of fibrin generation, when compared to the control group. 

### 3.3. HHALPCs Induce a Less Explosive Activation of Coagulation in an In Vitro Model with Whole Blood of Cirrhotic Patients

**Figure 3:**
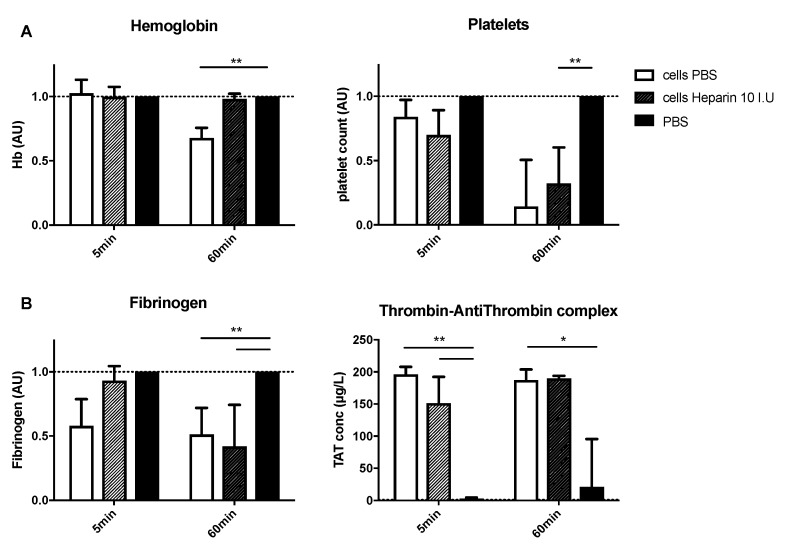
HHALPCs were added to the tubing loops, with or without added low dose heparin (10 IU). Analyses were performed after 5 and 60 min. (**A**,**B**) Values (except for TAT complex levels) were normalized compared to those of control tubing loops containing only PBS. Data are expressed as arbitrary units (AU). Bars represent medians with interquartile ranges (n = 6). Mann–Whitney test (* *p* < 0.05, ** *p* < 0.01).

Using the Chandler tubing loop model, blood samples from ADC patients (n = 6, ACLF grade 1) were exposed to HHALPCs, in order to detect procoagulant activity. The HHALPCs appeared to induce activation of the coagulation, but in a less explosive way compared to blood samples from healthy volunteers. Nevertheless, platelets and fibrinogen levels decreased significantly and TAT levels increased significantly after 1 h, confirming the activation of coagulation (Figure 3A,B). On the contrary, coagulation factors II, V and VIII were not as affected as in control blood samples (Supplementary Data S3). Concerning the anticoagulant pathways, only antithrombin levels decreased significantly, but no change in protein C or S was observed (Supplementary Data S4).

### 3.4. Infusion of a High Dose of HHALPCs Induces Coagulation and Alterations in Liver Blood Flow In Vivo

**Figure 4:**
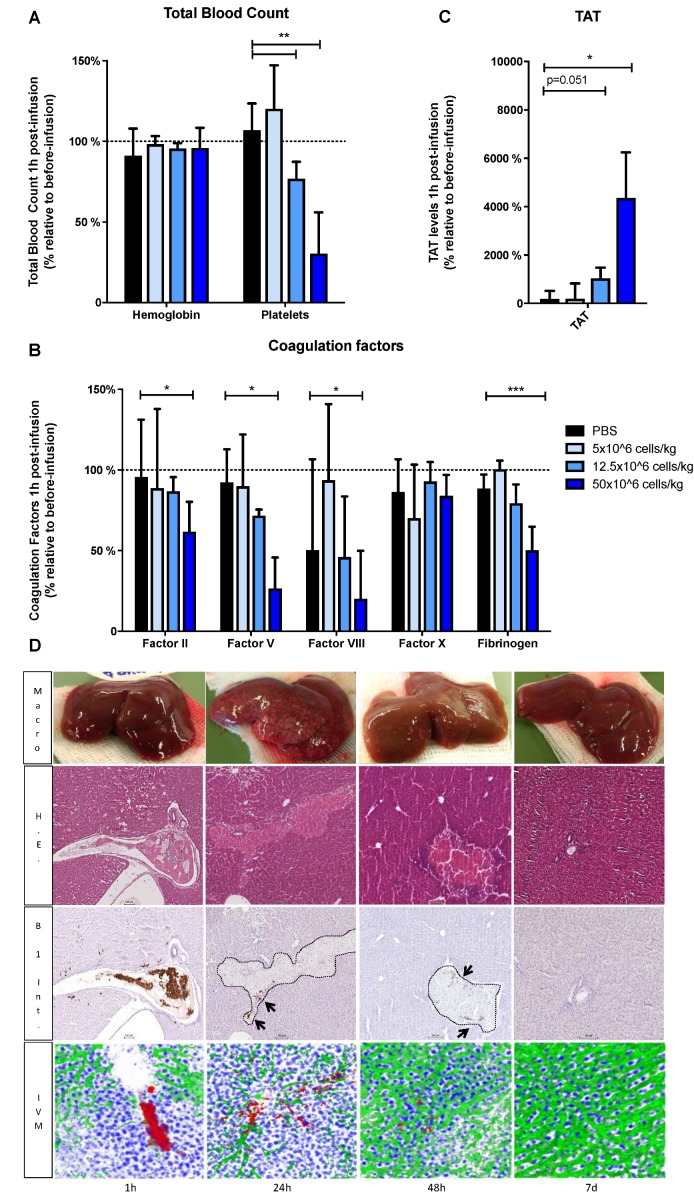
(**A**–**C**) Wistar rats were transplanted with different cell doses, 5 × 10^6^ cells/kg, 1.25 × 10^7^ cells/kg and 5 × 10^7^ cells/kg, by intraportal injection. Blood samples were taken before and at 1 h after transplantation. Total blood count, coagulation factors and TAT levels were analyzed and expressed (in %) relative to basal levels before cell infusion. Bars represent medians with interquartile ranges (n = 6/group). Mann–Whitney test (* *p* < 0.05, ** *p* < 0.01, *** *p* < 0.001). (**D**) Wistar rat were transplanted with the highest cell doses, 5 × 10^7^ cells/kg and analyzed at different time points (1 h, 24 h, 48 h and 7 days) by intravital microscopy (IVM) with objective 25× (n = 3/group). Z-stacks were collected to reconstruct 3D images. For IVM, HHALPCs were stained with Cell tracker red (red), and liver vasculature with Fluorescein isothiocyanate (FITC)-dextran (green) and cell nuclei with Hoechst 33342 (blue). Livers were harvested for pathological analysis. Hematoxylin eosin (H.E.) and human beta1 integrin staining (B1 Int.), to detect HHALPCs, were performed at the different time points (objective 10×).

To assess the effect of high dose infusion of HHALPCs, Wistar rats (n = 6/group) were infused with 5 × 10^7^ cells/kg, a dose previously used in clinical trials for patients suffering from liver-based metabolic diseases [3,21]. The high dose infusion was compared to intermediate (1.25 × 10^7^ cells/kg), and low (5 × 10^6^ cells/kg) cell doses. One h after HHALPCs infusion, platelet counts and coagulation factor levels were decreased in a dose-dependent manner. In particular, the high cell dose induced a significant decrease in platelet count, levels of fibrinogen and coagulation factors II, V and VIII, whereas it increased the TAT complex levels (Figure 4A–C). Hemoglobin and factor X levels were stable during cell infusion.

To assess hepatic flow disturbances related to the cell infusion, we performed intravital microscopy followed by immunohistochemistry on Wistar rats (n = 3/group), sacrificed at different time points after the infusion. Macroscopically, numerous discolored areas of liver tissue were detected after 24 h, however decreased after 48 h, and resolved after 7 days. Microscopically, large aggregates of HHALPCs appeared in the liver portal veins 1 h after infusion, likely causing blockage in the liver vasculature. The accumulation of cell aggregates was accompanied by a decreased FITC-dextran diffusion into the hepatic tissue, as observed by intravital microscopy. After 24 h of infusion, grouped infused cells were still present in liver sections near the vasculature. These cells were surrounded by large necrotic zones, as examined by HE staining (representative Videos S1 and S2 in Supplementary Data). After 48 h, the liver vascularization was improved with a clear regression of the necrotic zones and complete normalization was achieved after 7 days (Figure 4D).

### 3.5. Low Dose Infusion of HHALPCs In Vivo in Xenotransplant Rat Model without Thrombogenic Effect

The lowest dose of 5 × 10^6^ cells/kg which did not induce significant changes in platelet count, or in levels of coagulation factors or TAT complex (Figure 4), was not accompanied by noticeable alterations in liver sections. Identical results were observed when the same cell dose was infused peripherally (Supplementary Data S5).

### 3.6. Thrombogenic Effect Associated with the High Cell Dose Infusion In Vivo is Suppressed by Anticoagulant Drugs

**Figure 5:**
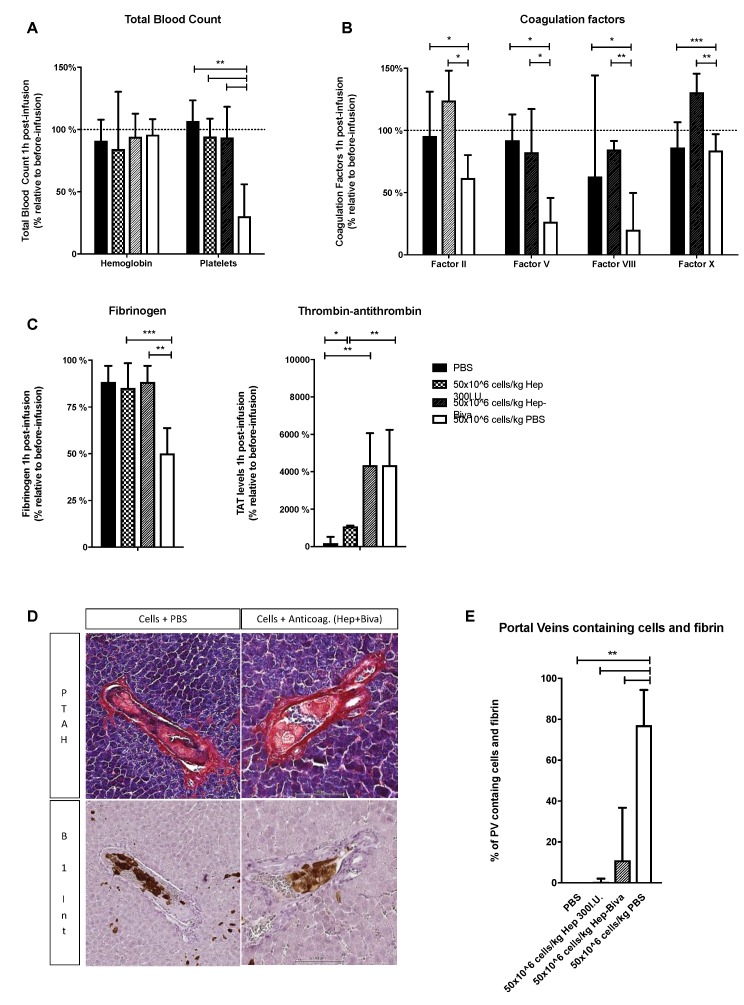
Wistar rats were transplanted with 5 × 10^7^ cells/kg HHALPCs, by intraportal injection, with or without (PBS) anticoagulant treatment, i.e., the combination of heparin and bivalirudin (hep-biva) or a high dose of heparin (hep 300 IU). (**A**–**C**) Blood samples were taken before and at 1 h after transplantation. Total blood count, coagulation factors and TAT levels were analyzed and were expressed relative to basal levels before cell infusion (in %). Bars represent medians with interquartile ranges (n = 6/group). Mann–Whitney test (* *p* < 0.05, ** *p* < 0.01, *** *p* < 0.001). (**D**) Liver sections were analyzed at 1 h after cell infusion by phospho-tungstic acid-hematoxylin stain (PTAH) and human beta1 integrin staining (B1 Int.). Objective 10×. (**E**) Quantification of the number of portal veins (PVs) that contained cells and fibrin, expressed as percentage of the total PVs containing cells. Bars represent medians with interquartile ranges (n = 6). Mann–Whitney test (** *p* < 0.01).

Since the infusion of a high cell dose appeared to induce local procoagulant activity, we studied how this could be suppressed by simultaneous application of anticoagulants. Two settings were compared; a combination of heparin (10 IU/5 × 10^6^ cells) and bivalirudin, previously investigated in our laboratory [17]; or a high dose of heparin (300 IU/5 × 10^6^ cells). Accordingly, Wistar rats (n = 6/group) were infused with 5 × 10^7^ cells/kg HHALPCs in the presence of one of the two anticoagulant arms above. Addition of heparin and bivalirudin significantly limited the decrease in platelet count, and the reduction in plasmatic fibrinogen, and factors II, V and VIII (Figure 5A–C). On the other hand, TAT complex levels were still increased (Figure 5B), suggesting that in vivo activation of the coagulation was not completely prevented. Addition of the higher heparin dose was more effective, limiting the drop in platelet count and fibrinogen level, and additionally reducing TAT complex levels (Figure 5A–C), compared to rats infused without anticoagulants. Again, hemoglobin levels were unchanged.

When examining liver sections from these rats on serial slices (Figure 5D) stained for fibrin and human cells (antibody against human beta1 integrin), we observed HHALPCs in the portal veins (PVs), surrounded by fibrin (blue strains). This pointed to HHALPC-induced thrombus formation. We quantified the numbers of portal veins containing HHALPCs and fibrin (Figure 5E). When the high cell dose was infused without anticoagulant, we detected both HHALPCs and fibrin in 80% (CI95%: 67–93%) of the portal veins. Infusion with anticoagulants heparin and bivalirudin resulted in positive staining in only 16% (CI95%: 3–35%) of the portal veins. Furthermore, no fibrin deposition was observed when the high dose of heparin was used during infusion.

## 4. Discussion

Therapy-induced thrombotic events are potential and major complications in MSC infusions [1,2,3,4,5,6]. Previous studies confirmed the expression of TF by HHALPCs [17]. Our study shows the important link between TF and thrombogenesis, when TF-expressing MSCs are exposed to human blood. In a whole blood perfusion assay, blocking of the TF coagulation pathway suppressed fibrin generation and platelet activation. 

Our study confirmed previous animal model data which showed that thrombotic events, induced by infusions of MSCs expressing TF, depend on the cell dose used [11]. In the present study, using high doses of MSCs infused through the intraportal route, we observed transient alterations in liver blood flow, with normalization of liver vasculature after 48 h. In contrast, we did not observe alterations of liver blood flow when lower cell doses were used. On the other hand, it is known that cell size can play a role in small vessel obstruction after cell therapy [39]. The higher the total number of cells infused, the more mechanical entrapment is possible, especially as the liver portal flow constitutes a terminal circulation. 

In the present rat study, blood analyses confirmed activation of the coagulation cascade in a cell dose-dependent manner, with significant decreases in platelets and levels of fibrinogen, factor II, V and VIII, and increases in TAT complex levels after infusion of higher cell dosage of 5 × 10^7^ cells/kg. The coagulation activation could however be prevented by two different anticoagulant protocols. Firstly, a combination of low doses of heparin (10 IU/5 × 10^6^ cells) and bivalirudin could limit the extent of the activation as previously described [17], i.e., by preventing the decrease in platelet count and factors II, V and VIII levels in rats. Since the procoagulant activity depended on the cell dose used, heparin was added to the cell suspension and amounts were adjusted according to multitudes of 5 × 10^6^ cells. Classic therapeutic heparin treatments in patients receiving MSC infusion was indeed shown not to be effective [40]. 

In the whole-blood tubing loop model however, the combination of anticoagulants did not prevent the activation of coagulation by HHALPCs, as seen by the residual increase in TAT complex levels in transplanted rats. High doses of heparin alone however, such as 300 IU/5 × 10^6^ cells, proved to control activation of coagulation by HHALPCs in whole blood. In vivo, this high heparin dose added to the infused cells prevented the decrease in platelets and fibrinogen, and limited the generation of TAT complex levels, thus suggesting a controlled activation of the coagulation process to a subclinical state. 

In clinical practice, due to their immunomodulatory and anti-fibrotic effects, low doses of HHALPCs could be used to treat ADC patients [23,24]. In the Chandler tubing loop model, HHALPCs seemed to activate the coagulation cascade of these patients in a less explosive way, inducing a decrease in platelet and fibrinogen levels only, without influencing coagulation factor levels II, V and VIII. Cirrhotic liver patients appear to have a “reset” hemostatic balance, due to simultaneous decreases in procoagulant and anticoagulant factors, usually designated as a “rebalanced hemostasis”. This “reset” balance is however more fragile than the balance in control blood and can thus alter more abruptly into either a prothrombotic or hemorrhagic condition [27,41]. This could explain why HHALPCs induce activation of coagulation in a less explosive way. Due to lower levels of both pro- and anticoagulant factors, identical amounts of TF on HHALPCs may trigger less explosive generation of thrombin, resulting in less fibrin formation and platelet activation, when compared to the control blood samples. Thus, infusions of HHALPCs in clinical trials could be safe for ADC patients, as long as platelets and fibrinogen levels are monitored closely.

Although anticoagulant drugs were found to modulate the procoagulant activity of HHALPCs, these may also influence the cell transplantation efficacy. The TF-linked procoagulant activity of progenitor cells can actually support cell engraftment [42,43] and stimulate liver regeneration [44]. Heparin could impair the homing capacity of the transplanted cells by interfering with the CXCR4/SDF-1 axis, decreasing cell implantation [43]. Especially for ADC cell infusions, a reduction of intrahepatic fibrin deposition by blocking the activation of the coagulation cascade could interfere with a helpful role in liver regeneration.

## 5. Conclusions

In conclusion, we demonstrated that TF, known to be expressed by HHALPCs [17], induces in a cell dose dependent manner, the activation of coagulation, resulting in fibrin generation and platelet activation. We confirmed that the thrombotic risk linked to HHALPCs infusion is related to the cell dose used. No modifications in blood parameters or liver vascularization were observed after infusions of low cell doses, such as 5 × 10^6^ cells/kg, as shown in control rats. The thrombogenic risk induced by infusions of a high cell doses, 5 × 10^7^ cells/kg, could be controlled by adding anticoagulant drugs, such as the combination of heparin and bivalirudin, or high doses of heparin alone. We finally showed, using a non-anticoagulated whole blood model, that HHALPC-induced activation of coagulation is less strong in ADC patients than for healthy control subjects. For clinical practice, this implies that lower cell doses (5 × 10^6^ cells/kg) may be used to treat ADC patients, while monitoring platelets and fibrinogen levels. On the other hand high cell doses in combination with anticoagulant drugs, could be used to treat patients presenting with liver-based metabolic disorders. Further clinical trials are needed to confirm these discoveries and study the effect of anticoagulation on cell implantation.

## Supplementary Materials

The following are available online at https://www.mdpi.com/2073-4409/8/8/846/s1, Figure S1: Animal preparation for the real-time study of liver vasculature by IVM after HHALPCs infusion, Figure S2: Coagulation factor X of tubing loops with HHALPCs in healthy control blood (n = 4), Figure S3: Blood parameters of tubing loops with HHALPCs in acute decompensated cirrhotic blood (n = 6), Figure S4: Blood parameters of tubing loops with HHALPCs in acute decompensated cirrhotic blood (n = 6) after 5 and 60 min, Figure S5: Intraportal infusion of HHALPCs in Wistar rats: infusion of 5 × 10^6^ cells/kg by intraportal and peripheral vein (n = 6/group), Table S1: Patient characteristics used for Chandler tubing loop experiments, Video S1-2: Intravital microscopy of the liver of a Wistar rat 24 h after intraportal infusion of 50 × 10^6^ cells/kg.
