# Supplementary material for: Clinical Protocol to Prevent Thrombogenic Effect of Liver-Derived Mesenchymal Cells for Cell-Based Therapies
Source: Cells. 2019 Aug 7;8(8):846. doi: 10.3390/cells8080846 (PMC6721739; doi:10.3390/cells8080846)
Supplement: Supplementary file 1 [file cells-08-00846-s001.zip › cells-555137-supplementary-english-layout-/supplementary data CELLS/Supplementary data.pdf]

Supplementary Data:

Table S1. Patient characteristics used for Chandler tubing loop experiments

	Controls (n=4)	ACLF grade 1 (n=6)
Age	35 ± 6	58 ± 16
Cause of cirrhosis (% of total)		
Alcoholic	n/a	60%
NASH	n/a	40%
MELD	n/a	24 ± 7
Hemoglobin (g/dL)	15.8 ± 0.5	8.7 ± 1.2
WBC (x10^3/µL)	6.1 ± 2.2	3.5 ± 1.2
Platelets (x10^3/µL)	239 ± 73	59 ± 27
Albumin (g/L)	n/d	34 ± 16
Creatinin (mg/dL)	n/d	2.58 ± 1.67
Bilirubin (mg/dL)	n/d	4.4 ± 6.6
AST (U/L)	n/d	132 ± 186
ALT (U/L)	n/d	85 ± 117
GGT (U/L)	n/d	158 ± 186
INR	n/d	1.46 ± 0.31
Fibrinogen (mg/dL)	287 ± 116	212 ± 66
Antithrombin (%)	n/d	35 ± 15
Protein S (%)	97 ± 9	68 ± 23
Protein C (%)	89 ± 11	48 ± 20
Factor II (%)	95 ± 5	41 ± 19
Factor V (%)	72 ± 19	35 ± 19
Factor VIII (%)	73± 22	171 ± 60
Factor X (%)	72 ± 11	57 ± 17

ALT: alanine transaminase; AST: aspartate transaminase; GGT: gamma-glutamyl transpeptidase; INR: international normalized ratio; MELD: model for end-stage liver disease; n/a: not applicable; n/d: not determined; NASH: non-alcoholic steato-hepatitis; WBC: white blood count. Shown are means ± SD

Figure S1

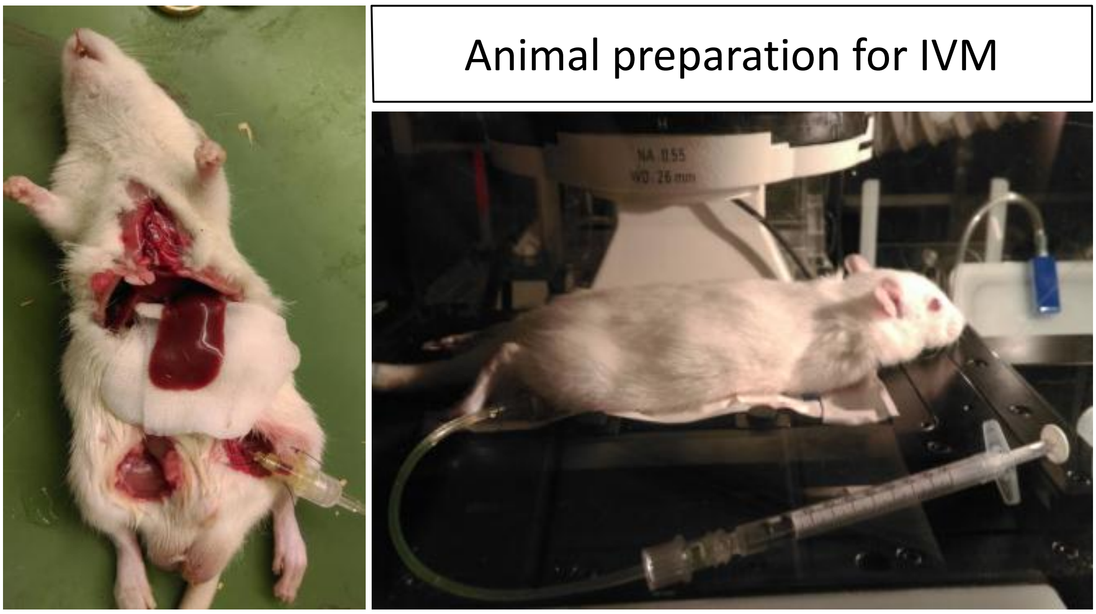

Figure S1. Animal preparation for the real-time study of liver vasculature by IVM after HHALPCs infusion. During IVM, animals were anesthetized by a combination of ketamine (80 mg/kg) and xylazine (10 mg/kg) by intraperitoneal injection. First, an intravenous access was assured by placing a femoral catheter (24G BD-Insyte-N 381311). Subsequently, Hoechst 33342 was injected to stain cell nuclei. Then, the left liver lobe was exposed, and the rat was placed in a prone position on the self-made coverslip dish and put on the microscope stage in a preheated 37 ℃ chamber. To assess the liver vasculature, 5 mg of FITC-dextran 70kDa (46945, Sigma) was injected via the femoral catheter.

Figure S2

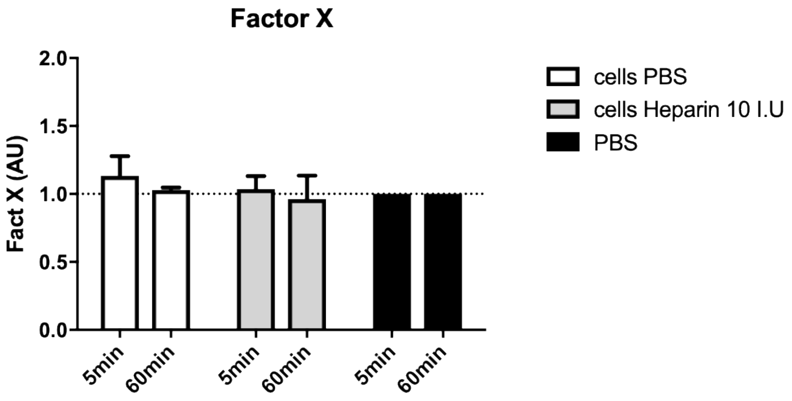

Figure S2. Coagulation factor X of tubing loops with HHALPCs in healthy control blood (n=4). Tubing loops were added with HHALPCs, with or without anticoagulant drugs, such as low dose heparin (10 IU), heparin and bivalirudin and high dose heparin (300 IU), as indicated. Measurements were performed after 5 and 60 min. Results were normalized with control tubing loops containing only PBS expressed as AU. Bars represent median with interquartile range. No significant difference was observed for factor X levels at 5 or 60 min, compared to tubing loops containing only PBS.

Figure S3

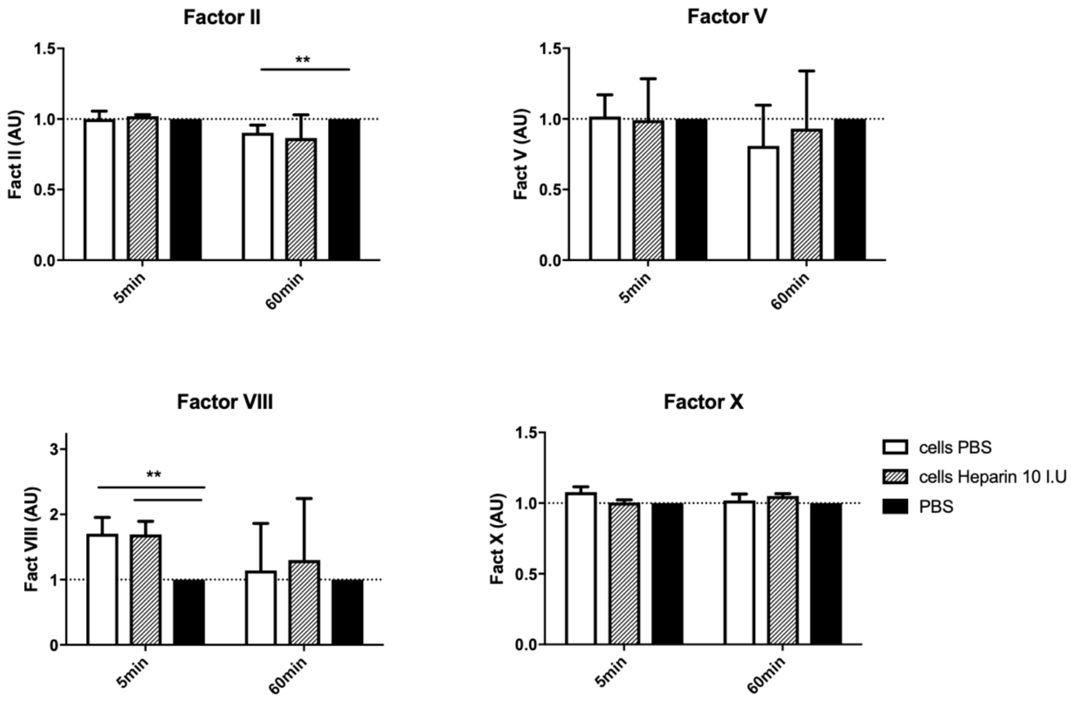

Figure S3. Blood parameters of tubing loops with HHALPCs in acute decompensated cirrhotic blood (n=6). Tubing loops were supplemented with HHALPCs in the presence or absence of a dose of heparin (10 I.U). Blood samples were taken after 5 and 60 min. Values were normalized in comparison to control tubing loops containing only PBS, and were expressed as AU. Bars represent medians with interquartile range. Mann-Whitney test. In cirrhotic blood, no consistent decrease was observed for factors II, V and VIII after 60 min, when compared to control blood. In contrast, factor X was stable in all samples.

Figure S4

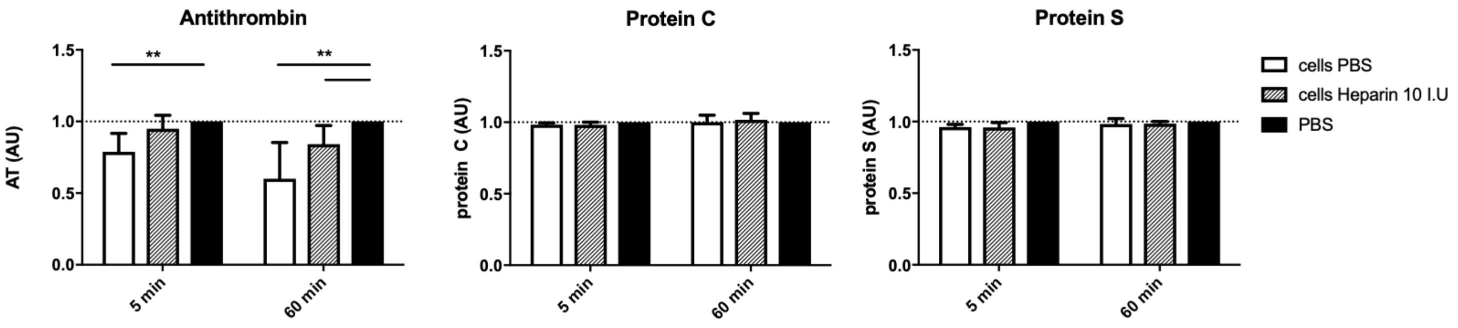

Figure S4. Blood parameters of tubing loops with HHALPCs in acute decompensated cirrhotic blood (n=6) after 5 and 60min. Analysis of the anticoagulation factors showed only a significant decrease for antithrombin after 60 min. No changes in protein C or S levels were observed.

Figure S5

A.

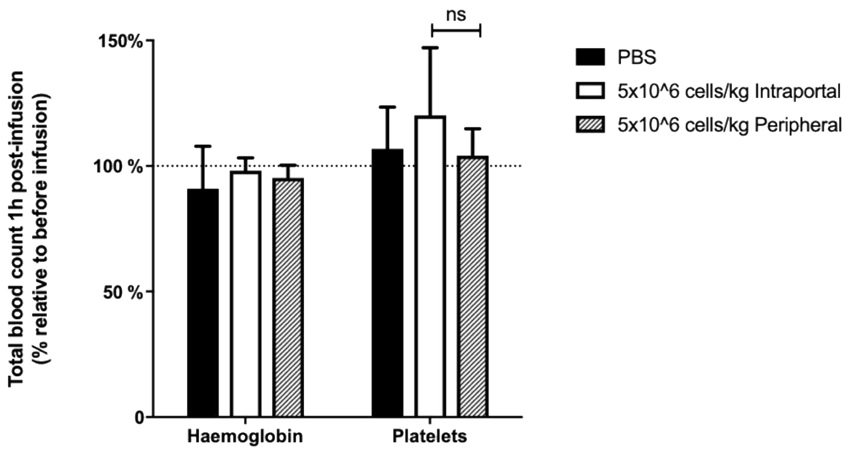

B.

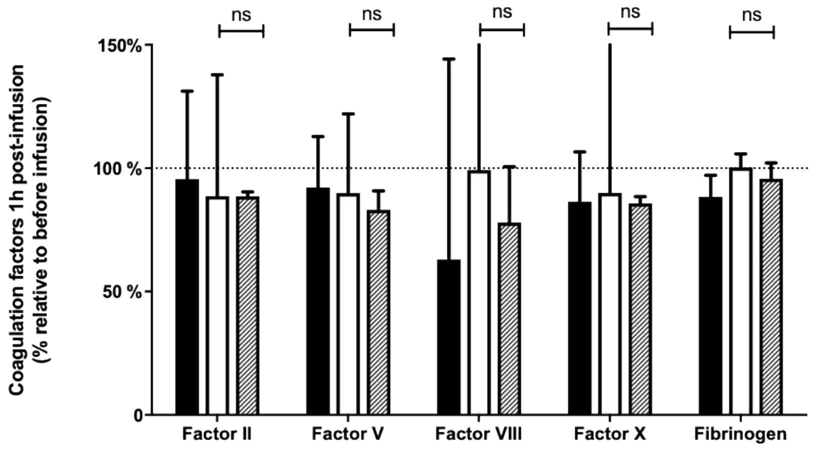

Figure S5. Intraportal infusion of HHALPCs in Wistar rats: infusion of $5x10^6$ cells/kg by intraportal and peripheral vein (n=6/group). Blood samples were taken before and at 1 h after transplantation. Total blood count and coagulation factors were analyzed and were expressed relative to basal levels before cell infusion (in %). Bars represent medians with interquartile range. Mann-Whitney test (*p<0.05, **p<0.01, ***p<0.001).

Video 1-2. Intravital microscopy of the liver of a Wistar rat 24h after intraportal infusion of $50x10^6$cells/kg. Microscope LSM510 Time lapse (1 image/15s, Obj. 25x). (1) After 24 h, HHALPCs stained with cell tracker (red) can be seen in the liver sinusoids, interfering with the surrounding liver vasculature stained with FITC-Dextran (green). Nuclei were counterstained with Hoechst 33342 (blue). (2) When only PBS is infused, liver vasculature is not altered with a good visualization of liver sinusoids by FITC-dextran staining (green). Nuclei were counterstained with Hoechst 33342 (blue).
